# The impact of negative urgency on implicit mobile phone addiction tendency among college freshmen in the context of social exclusion

**DOI:** 10.3389/fpsyg.2024.1426450

**Published:** 2024-07-08

**Authors:** Wei Liu, Mengmeng Zhao, Ruixin Wang, Manxi Yang, Zhenqiang Zhang, Shaogang Song, Lina Li

**Affiliations:** ^1^College Student Mental Health Education and Counseling Center, North China University of Science and Technology, Tangshan, China; ^2^School of Psychology and Mental Health, North China University of Science and Technology, Tangshan, China; ^3^Student Affairs Office, Caofeidian College of Technology, Tangshan, China; ^4^School of Aeronautics and Astronautics, Caofeidian College of Technology, Tangshan, China

**Keywords:** negative urgency, social exclusion, implicit attitude, mobile phone addiction tendency, college freshmen

## Abstract

**Purpose:**

The purpose of this study is to investigate the impact of negative urgency on implicit mobile phone addiction tendency among college freshmen, and to observe whether social exclusion situations affect the relationship between negative urgency and implicit mobile phone addiction tendency.

**Methods:**

The UPPS-P Impulsive Behavior Scale was used to screen 575 freshmen from a certain university. The experiment utilized a GO/NO-GO paradigm. Experiment 1 employed a 2 (negative urgency group: high negative urgency group, low negative urgency group) × 2 (word type: phone related words, phone non-related words) two-factor mixed experimental design. Experiment 2 employed a 2 (negative urgency group: high negative urgency group, low negative urgency group) × 2 (social exclusion type: priming group, non-priming group) × 2 (word type: phone related words, phone non-related words) three-factor mixed experimental design.

**Results:**

Experiment 1 results showed a significant main effect of negative urgency group and a significant interaction effect between negative urgency group and word type. Experiment 2 results demonstrated a significant main effect of negative urgency group and a significant main effect of social exclusion type. There was a significant interaction effect between word type and social exclusion type, as well as between word type and negative urgency group. The three-way interaction effect among negative urgency group, word type, and social exclusion type was significant.

**Conclusion:**

College freshmen with high negative urgency exhibit a higher tendency toward implicit mobile phone addiction. In social exclusion situations, college freshmen show a higher tendency toward implicit smartphone addiction. Social exclusion situations and negative urgency jointly influence the implicit mobile phone addiction tendency of college freshmen.

## Introduction

In the contemporary information era, mobile phones have become the most widely used internet access tool due to their convenience and accessibility. As of June 2021, the scale of mobile internet users in China reached 1.07 billion, which means that 99.6% of internet users utilize mobile phones to go online (China Internet Network Information Center, 2021). However, while mobile phones bring convenience to people’s lives, they also pose the risk of addiction (He et al., 2012; Barnes et al., 2019). Mobile Phone Addiction (MPA), also known as Problematic Mobile Phone Use (PMPU), refers to a behavioral addiction in which individuals experience significant impairment in their psychological and social functioning due to excessive and uncontrollable mobile phone use (Liu et al., 2017). Research findings indicate that mobile phone addiction can have adverse effects on university students’ academic performance and quality of life (Tian et al., 2021; Yang et al., 2022), and is also closely associated with individuals’ experiences of negative emotions such as anxiety and depression (Mahsa et al., 2023; Niu, 2023).

### Mobile phone addiction tendency of college freshmen

College students are a high-risk and susceptible group for mobile phone addiction, which has shown a continuous upward trend in recent years (Peng et al., 2022). College students are at a critical stage of identity formation, role adjustment, and psychological maturation (Janet et al., 2018), and through the interaction of internal and external factors, students of different grade levels may develop distinct identity formations (Zhu and Qi, 2018). Under the influence of these varying identities, individuals exhibit diverse adaptive social behaviors (Oyserman, 2009). Freshmen university students face numerous challenges, such as changes in the learning environment and the increased complexity of interpersonal relationships. If these students have poor adaptive abilities and cannot actively cope with these challenges, some may turn to mobile phone use as a means of seeking a sense of belonging and emotional refuge, potentially leading to the development of mobile phone addiction (Tian and Wang, 2023). For instance, Zhao et al. (2023) examined the grade-level differences in the formation of smartphone addiction among female university students, finding that for lower-grade students, their school adaptation ability can predict the development of mobile phone addiction, compared to their higher-grade counterparts.

### Implicit mobile phone addiction tendency

Greenwald and Banaji (1995) proposed the concept of implicit social cognition, revealing the involvement of unconscious components in conscious social cognition processes. The Dual Attitude Model (DAM) (Wilson et al., 2000) posits that individuals may concurrently hold both explicit and implicit attitudes toward the same attitude object. Implicit attitudes are typically automatically activated, while explicit attitudes require more psychological energy and motivation to retrieve from the memory system. When individuals access their explicit attitudes and their intensity surpasses and suppresses the implicit attitudes, they report and exhibit the explicit attitudes, concealing the implicit attitudes. Conversely, when individuals lack cognitive energy and motivation to retrieve their explicit attitudes, they can only report their implicit attitudes. Importantly, research indicates that self-report methods are insensitive to implicit thoughts or those occurring beyond consciousness (Derbyshire and Keay, 2023). The paradigm of implicit cognition does not necessitate participants to directly report their feelings, thereby mitigating to some extent the impact of social desirability bias (where participants may be influenced to provide socially acceptable responses). This approach can offer more authentic implicit attitude information, aiding researchers in gaining deeper insights into individuals’ unconscious biases and emotional predispositions.

Research into implicit cognition can aid our comprehension of why individuals persistently engage in addictive behaviors despite being aware of the adverse consequences (Stacy and Wiers, 2010). Implicit cognition refers to the automatic thoughts that are not easily perceived or controlled by an individual. When exposed to cues related to addiction, whether through personal recollection or visual stimuli, an individual’s attention is invariably drawn to the addictive object or behavior. During the development of addiction, a person’s attitudes are subconsciously influenced, guiding them to select addictive behaviors as a means of coping with an inhospitable environment (Nock et al., 2010). This often results in the formation of a positive preference for addictive stimuli (Rudman, 2004), which may not be consciously desired or recognized by the individual. Such cognitive biases can exacerbate the development and persistence of addictive behaviors. In essence, implicit associative cognition is a significant cognitive bias that leads to addictive behaviors (Turel and Serenko, 2020).

This study employed a modified GO/NO-GO paradigm to assess individuals’ implicit mobile phone addiction tendency. The “NO-GO” condition was used to instruct participants to inhibit their response to a specific stimulus, with participants’ accuracy reflecting their inhibitory control over the stimulus. A higher accuracy rate indicates stronger inhibitory control (Wang et al., 2024). In other words, when assessing individuals’ implicit attitudes toward mobile phone addiction, a higher error rate in responding to mobile phone-related words under the “NO-GO” condition indicates an inevitable response to the phone-related stimuli, an inability to control the processing of phone-related information, and thus an implicit mobile phone addiction tendency.

### Negative urgency and mobile phone addiction

Based on the biopsychosocial model of behavioral addiction, Wang et al. (2013) have posited that psychological factors are significant contributors to the susceptibility and maintenance of behavioral addictions, with personality traits being one of the primary pathological factors leading to such addictions. Impulsive personality has been identified as a robust predictor of mobile phone addiction (Mei et al., 2017; Sullivan et al., 2021; Ji et al., 2023). Impulsivity is a multidimensional concept, with negative urgency being a pivotal dimension, which can be construed as the intense and urgent desire to seek immediate gratification when faced with stress, discomfort, or the desire to avoid pain. This sense of urgency often compels individuals to engage in irrational and reckless behaviors, despite the potential for long-term adverse consequences (Anestis et al., 2009). Negative urgency is closely associated with addictive behaviors (Kyoung and Hyejeen, 2019), as such behaviors are frequently employed to escape or alleviate negative emotions. There is research to prove that negative urgency is the best predictor of substance abuse behavior (Littlefield et al., 2015). Consequently, it is hypothesized that negative urgency may be a risk factor influencing the propensity for mobile phone addiction.

### Social exclusion, negative urgency, and mobile phone addiction

Social exclusion refers to the phenomenon where an individual is ignored, ostracized, and rejected by a social group or other individuals, thereby hindering their need for belonging and relationships (Roz, 2007). Negative emotions are often the direct consequence of social exclusion (Randy et al., 2024; Shuai et al., 2024). When faced with exclusionary situations, individuals may experience intense and urgent impulses to take immediate action to resolve or avoid the issue, thereby triggering or intensifying a sense of negative urgency (Horváth et al., 2022). Furthermore, previous studies have indicated a correlation between social exclusion and internet addiction (Douglas et al., 2008). The social compensation theory of addiction suggests that when individuals feel socially excluded and lack recognition and a sense of belonging, their social needs are unmet. The internet provides an environment suitable for compensation, leading to an increase in internet usage and the emergence of addictive behaviors (Kraut et al., 2002). For college freshmen, transitioning from the intense study environment and relatively simple interpersonal relationships of high school to the more frequent and extensive social interactions of college increases their social needs. When individuals struggle to adapt to their new environment, they may experience social exclusion, which could lead them to compensate through increased mobile phone usage.

In summary, utilizing a modified GO/NO-GO paradigm, experiment 1 investigates the impact of negative urgency on the implicit mobile phone addiction tendency among college freshmen. Experiment 2 explores whether the inclusion of environmental factors, specifically social exclusion scenarios, alters the relationship between negative urgency and implicit mobile phone addiction tendency. The study aims to elucidate the role of negative urgency in implicit mobile phone addiction tendency and the influence of social exclusion contexts on this relationship. From this, we propose the following hypotheses: H1: Individuals with high negative urgency, compared to those with low negative urgency, exhibit a greater implicit mobile phone addiction tendency, which is reflected in a lower accuracy rate on NO-GO tasks involving phone related words; H2: Compared to situations without social exclusion, individuals with high negative urgency exhibit a greater implicit mobile phone addiction tendency in situations of social exclusion, which is indicated by a lower accuracy rate in NO-GO tasks for phone related words under situations of social exclusion. By experimentally examining the connection between negative urgency and implicit mobile phone addiction tendency, we can gain a deeper understanding of the interplay between personality traits and environmental factors in the genesis of mobile phone addiction. This not only contributes to a more profound comprehension of the mechanisms underlying mobile phone addictive behaviors but also provides significant guidance for future intervention and prevention efforts.

## Participants and methods

### Participants

#### Experiment 1

A convenience sampling method was employed to select 575 freshmen from a certain university and distribute the UPPS-P Impulsive Behavior Scale. A total of 560 valid questionnaires were returned, yielding a response rate of 97.4%. The average age of the participants was 18.62 ± 0.82 years (ranging from 17 to 22 years old). Using the extreme groups approach, the scores of the negative urgency scale were sorted in descending order. Referring to previous research (Duan et al., 2020), participants whose scores fell within the top and bottom 27% were selected. A total of 80 participants were randomly chosen, with 40 in the high negative urgency group (16 males, 24 females, age 18.64 ± 0.81) and 40 in the low negative urgency group (25 males, 15 females, age 18.62 ± 0.79). An independent samples *t*-test was conducted. The results indicated a significant difference in negative urgency scores between the high and low groups (*p* < 0.001). Four participants did not attend or withdrew from the experiment midway, resulting in final group sizes of 39 for the high negative urgency group and 37 for the low negative urgency group.

#### Experiment 2

The selection of participants was identical to that of Experiment 1. A total of 80 individuals were selected for the high negative urgency group and another 80 for the low negative urgency group. Subsequently, participants were grouped according to the ABBA balanced method based on their scores, with 80 individuals undergoing social exclusion priming and another 80 receiving non-social exclusion priming. Participants for whom the social exclusion priming was ineffective were excluded from the study. Ultimately, the number of participants in each group was determined as follows: 34 in the high negative urgency-priming Group, 34 in the high negative urgency-non-priming group; 33 in the low negative urgency-priming group, and 36 in the low negative urgency-non-priming group.

Before the formal experiment, an interview was conducted with all subjects to ensure that they had normal vision or corrected vision, no visual or auditory impairment, and to ensure that the subjects had not participated in similar experiments in the past month. The subjects were also made to understand that the purpose of this study was the measurement of keystroke response time, after which the principle of voluntariness was completely followed and the subjects willing to participate in the experiment were asked to sign an informed consent form.

### Measures

#### The simplified UPPS-P impulsive behavior scale

It was revised in Chinese by Xue et al. (2017) and consists of 20 items across five dimensions: Negative Urgency, Positive Urgency, Lack of Perseverance, Sensation Seeking, and Lack of Premeditation. The scoring system for this scale ranges from “Very Inconsistent” to “Very Consistent,” with four points. In this study, only the four items pertaining to the negative urgency dimension were utilized. The Cronbach’s alpha coefficients for this scale in this study were above 0.67.

#### GO/NO-GO task materials

The stimuli were divided into two categories: one unrelated to mobile phones and the other related to mobile phones. The unrelated stimuli included words such as “hat,” “kitten,” “table,” and “chair.” The mobile phone-related stimuli were selected by five graduate students majoring in psychology from BaiduWenku, resulting in a list of 150 words. Subsequently, four graduate students with over 3 years of mobile phone usage experience in psychology rated the relevance of these words on a five-point scale. A score of 1 indicated very irrelevant, 2 somewhat irrelevant, 3 neutral, 4 somewhat relevant, and 5 very relevant. Ultimately, 100 words with an average score above 4 were chosen as mobile phone-related stimuli.

#### The social exclusion induction tool

This study employs the Cyberball paradigm by Williams et al. (2000) to elicit a sense of social exclusion in individuals. Prior to the experiment, participants are informed that they will engage in a ball-tossing game with two other individuals, and they are instructed to imagine the scenario as vividly as possible, including the appearance and expressions of their peers, as well as the ambient conditions. The two other players are fictitious, and their participation is pre-programmed to control the frequency with which the actual participant receives the ball. Over the course of 30 tosses in the game, the priming group receives the ball only during the initial two rounds and is subsequently excluded from further play; the non-priming group, however, receives the ball more than 10 times, exceeding a third of the total tosses.

### Procedures

This study draws upon the variant of the classic GO/NO-GO task as referenced by Kreusch et al. (2014), utilizing phone related words and phone non-related words as stimuli to explore participants’ inhibitory control abilities regarding phone related words, thereby further predicting their implicit mobile phone addiction tendency.

#### Experiment 1

The GO/NO-GO task is scripted using E-prime 2.0 psychological software on a Hewlett-Packard computer with a resolution of 1,920 × 1,080 to present stimulus materials. The task is divided into two main sections: practice and the formal experiment, with stimuli consisting of the letter’s “O”/“E” and mobile-related/unrelated vocabulary. During the practice phase, participants are instructed to press the spacebar as quickly and accurately as possible when the letter “E” appears and to refrain from pressing the key when the letter “O” appears, with feedback provided. This phase includes 20 trials, with the letter “E” appearing five times, ensuring that participants fully understand the experimental process and can operate correctly before proceeding to the formal experiment. The results of the practice phase are not included in the final score. The formal experiment comprises two parts: one part requires participants to respond to phone related words and not to unrelated words; the second part is the inverse, requiring participants to respond to unrelated words and not to related words. The experiment was conducted with inter-subject balance.

#### Experiment 2

Conducted in a tranquil and well-lit psychology laboratory, the experimenter thoroughly explained the procedures prior to the commencement of the study to ensure that participants fully understood the experimental protocol. After participants were grouped, they engaged in the classic ball-tossing paradigm from the social exclusion scenario. Initially, instructions for the Cyberball task (CP) were presented on a white screen, stating that the game was designed to exercise participants’ imaginative abilities in preparation for subsequent experiments. Participants were instructed not to overly concern themselves with their performance but to focus on visualizing the game scenario, including the appearance and emotions of the other players, with as much realism as possible.

Upon the conclusion of the game, participants were asked to complete two questions: “Did you feel excluded during the game?” and “Did you feel ignored during the game?” These were rated on a scale from 0 (no feeling at all) to 10 (very profound feeling), following the method established by Xu et al. (2017). These questions served as a measure of the effectiveness of the social exclusion priming, with higher average scores indicating a greater sense of social exclusion. According to the criteria, the top 27% (a score of 7) is considered effective for social exclusion priming, while the bottom 27% (a score of 3) is deemed effective for non-social exclusion priming set (Gonsalkorale and Williams, 2007). In this study, the correlation coefficient between the two questions was *r* = 0.94 (*p* < 0.001). After responding to these questions, participants proceeded with the smartphone-related GO/NO-GO task identical to that in Experiment 1.

### Statistical methods

#### Experiment 1

A two-factor mixed experimental design was employed, consisting of 2 (negative urgency group: high negative urgency group, low negative urgency group) × 2 (word type: phone related words, phone non-related words). The negative urgency group served as the between-subjects variable, while word type acted as the within-subjects variable, with the NO-GO accuracy rate as the dependent variable. Experiment 2: A three-factor mixed experimental design was utilized, with 2 (negative urgency group: high negative urgency group, low negative urgency group) × 2 (social exclusion type: priming group, non-priming group) × 2 (word type: phone related words, phone non-related words). Here, the negative urgency group and social exclusion were the between-subjects variables, and word type was the within-subjects variable, with the NO-GO accuracy rate again serving as the dependent variable.

The lower the accuracy rate on phone related words in the NO-GO task, the weaker the individual’s inhibitory capacity toward phone-related stimuli, indicating a higher tendency for addiction. The final data were subjected to descriptive statistical analysis and repeated measures ANOVA, with statistical significance determined by a *p* value of less than 0.05.

## Results

### Experiment 1: analysis of NO-GO correct rates

**Figure 1:**
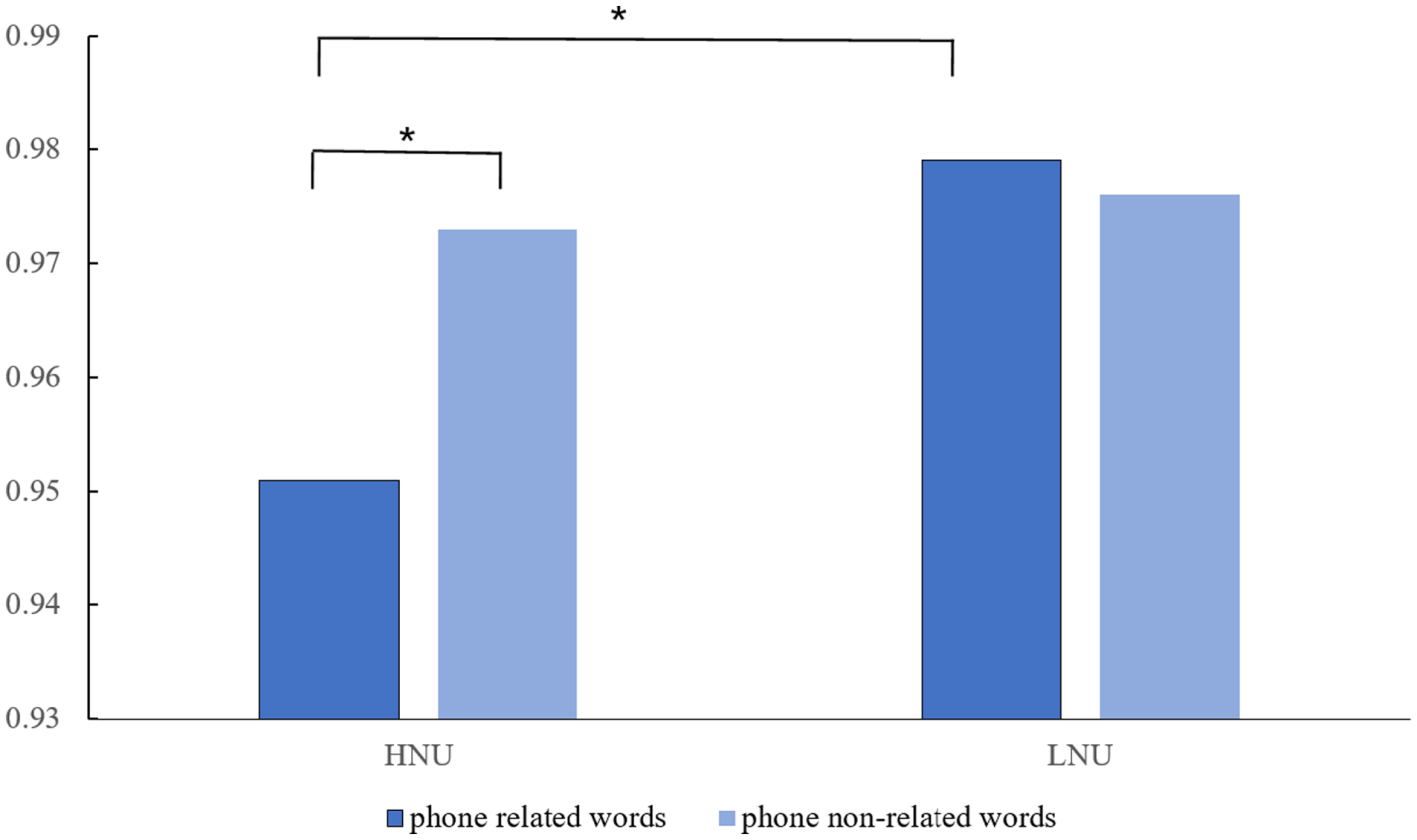
NO-GO accuracy rate of high and low negative urgency groups on phone-related words and phone non-related words. HNU, High negative urgency group; LNU, Low negative urgency group.

A repeated measures ANOVA was conducted on the dependent variable, the NO-GO accuracy rate. The results indicated a significant main effect of the negative urgency group [*F* (1, 74) = 6.701, *p* = 0.012, η^2^ = 0.083], with the high negative urgency group exhibiting a significantly lower NO-GO accuracy rate compared to the low negative urgency group. The main effect of word type was not significant (*p* > 0.05). However, a significant interaction effect was observed between the negative urgency group and word type [*F* (1, 74) = 4.164, *p* = 0.045, η^2^ = 0.053]. Further simple effect analysis revealed that the high negative urgency group showed a significant difference in word type (*F* = 6.970, *p* = 0.010, η^2^ = 0.086), specifically, the high urgency group’s NO-GO accuracy rate for phone related words was significantly lower than that of the low negative urgency group. For detailed information, refer to Table 1 and Figure 1.

**Table 1:** ANOVA on the accuracy of NO-GO for word types with different negative urgency group (*x* ± *s*).

Negative urgency group	Word type	Accuracy rate	F	p
High negative urgency group	Phone related words	0.951 ± 0.007	6.970	0.010
	Phone non-related words	0.973 ± 0.004		
Low negative urgency group	Phone related words	0.979 ± 0.008	0.077	0.782
	Phone non-related words	0.976 ± 0.004		

NUG, Negative urgency group; HNUG, High negative urgency group; LNUG, Low negative urgency group; WT, Word type; PRW, Phone related words; PNRW, Phone non-related words.

### Experiment 2: analysis of NO-GO correct rates

**Figure 2:**
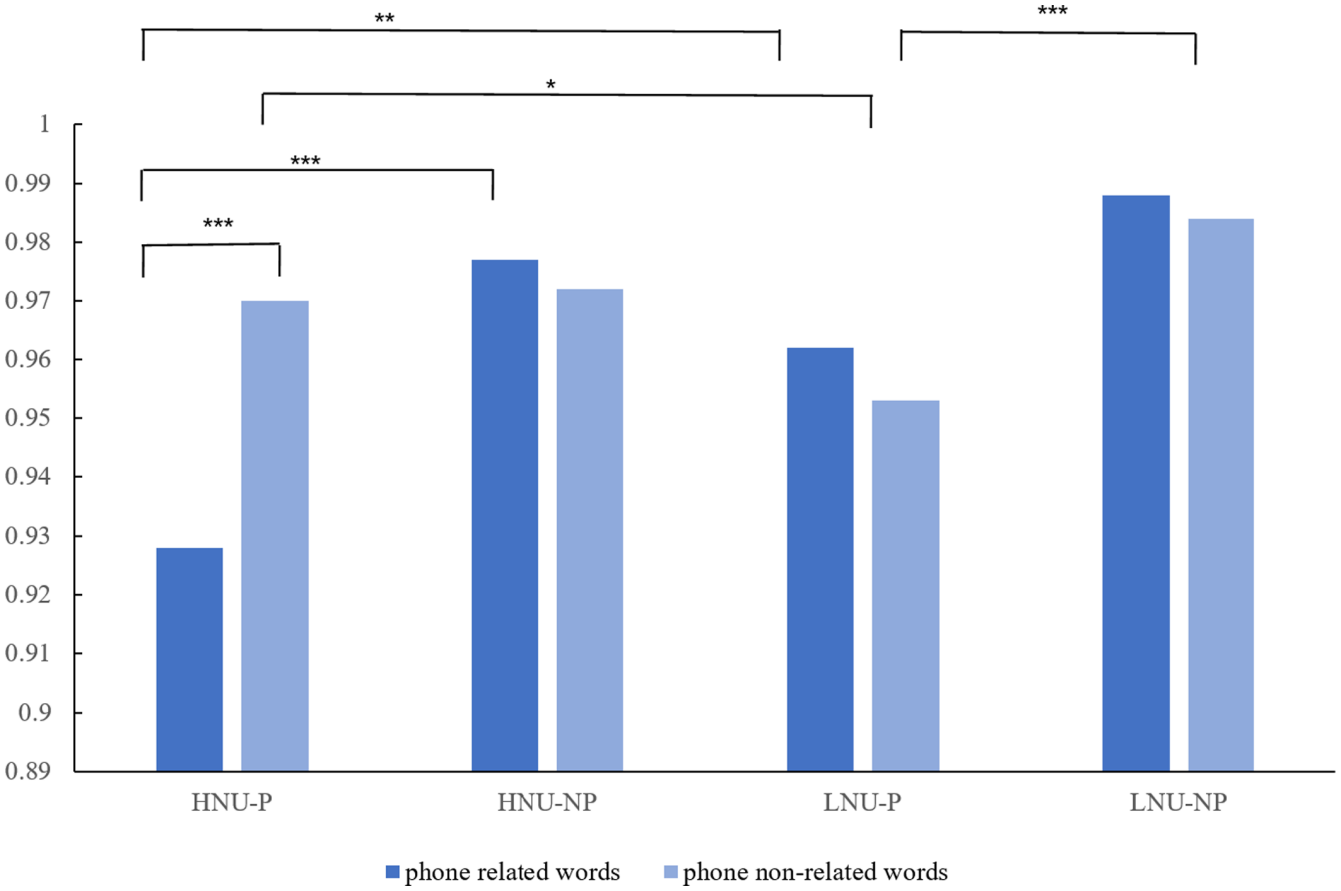
The NO-GO accuracy rate of different negative urgency and social exclusion in phone related words and phone non-related words. HNU-P, High Negative urgency-priming group; HNU-NP, High negative urgency-non-priming group; LNU-P, Low negative urgency-priming group; LNU-NP, Low negative urgency-non-priming group.

A repeated measures ANOVA was conducted on the dependent variable, the NO-GO accuracy rate. The findings revealed a significant main effect for the negative urgency group [*F* (1, 133) = 4.060, *p* = 0.046, η^2^ = 0.013], with participants in the high negative urgency group demonstrating a significantly lower NO-GO accuracy rate compared to those in the low negative urgency group. Additionally, a significant main effect was observed for the social exclusion type [*F* (1, 133) = 30.693, *p* < 0.001, η^2^ = 0.188], indicating that participants in the priming group had a significantly lower NO-GO accuracy rate than those in the non-priming group. The main effect of word type was not significant (*p* > 0.05). The interaction between the negative urgency group and word type was significant [*F* (1, 133) = 7.798, *p* = 0.006, η^2^ = 0.055]. Simple effects analysis indicated that the high negative urgency group was a significant difference based on word type (*F* = 7.840, *p* = 0.006, η^2^ = 0.055), with the high negative urgency group showing a notably lower NO-GO accuracy rate for phone related words than phone non-related words. This difference was not significant for the low negative urgency group (*p* > 0.05). The interaction between social exclusion type and word type was also significant [*F* (1, 133) = 5.003, *p* = 0.027, η^2^ = 0.036]. Simple effects analysis showed that the priming group was a significant difference based on word type [*F* (1, 133) = 5.961, *p* = 0.016, η^2^ = 0.042], with the priming group having a lower NO-GO accuracy rate for phone related words compared to phone non-related words. This difference was not significant for the non-priming group (*p* > 0.05). Furthermore, a significant three-way interaction among negative urgency group, social exclusion type, and word type was found [*F* (1, 133) = 8.304, *p* = 0.005, η^2^ = 0.059]. Simple effects analysis within this interaction showed that participants in the high negative urgency-priming group had a significantly lower NO-GO accuracy rate for phone-related words than for phone non-related words. Within the priming group, the high negative urgency group had a significantly lower NO-GO accuracy rate for phone-related words compared to the low negative urgency group (*p* = 0.004), while the high negative urgency group had a significantly higher NO-GO accuracy rate for phone non-related words compared to the low negative urgency group (*p* = 0.014) (Figure 2; Tables 2, 3).

**Table 2:** Results of NO-GO accuracy for negative urgency group under different social exclusion conditions (*x* ± *s*).

WT	SET	HNUG	LNUG
PRW	PG	0.928 ± 0.086	0.962 ± 0.027
	NPG	0.977 ± 0.026	0.988 ± 0.014
PNRW	PG	0.970 ± 0.025	0.953 ± 0.038
	NPG	0.972 ± 0.028	0.984 ± 0.017

HNUG, High negative urgency group; LNUG, Low negative urgency group; SET, Social exclusion type; WT, Word type; PRW, Phone related words; PNRW, Phone non-related words; PG, Priming group; and NPG, Non-priming group.

**Table 3:** ANOVA in response to word types in the negative urgency group and the social exclusion type.

Source of variation	MS	F	P	η^2^
NUG	0.007	4.06017	0.046	0.030
SET	0.050	30.693	<0.001	0.188
WT	0.002	1.800	0.182	0.013
NUG × WT	0.011	7.798	0.006	0.055
SET × WT	0.007	5.003	0.027	0.036
NUG × SET	<0.001	0.076	0.783	0.001
NUG × WT × SET	0.011	8.304	0.005	0.059

NUG, Negative urgency group; SET, Social exclusion type; WT, Word type.

## Discussion

The implicit mobile phone addiction tendency is primarily assessed by comparing the accuracy of individuals in the NO-GO condition with phone-related and non-related words. The lower the accuracy of phone-related words, the weaker the individual’s inhibitory control over them (Wang et al., 2024), indicating a higher implicit mobile phone addiction tendency. The dual-process model of addictive behavior posits that addictive behaviors are influenced by the interaction of impulsive precursors (implicit cognition), reflective precursors (explicit cognition), and limitations (Hofmann et al., 2008). Previous studies have predominantly focused on explicit attitudes toward addiction; hence, this study delves into the implicit mobile phone addiction of college freshmen from the perspectives of personality traits and environmental factors. Experiment 1 explores the impact of negative urgency on the implicit mobile phone addiction tendency of college freshmen from a personality standpoint, while Experiment 2 further investigates whether social exclusion scenarios can alter the influence of negative urgency on this addiction. The findings reveal that college freshmen with higher levels of negative urgency exhibit a greater implicit mobile phone addiction tendency compared to those with lower levels; under social exclusion scenarios, the implicit addiction tendency is heightened; and both social exclusion and negative urgency jointly affect the implicit mobile phone addiction tendency of college freshmen.

The results of Experiment 1 indicate that the main effect of negative urgency group is significant, and the interaction between negative urgency group and word type is also significant. Freshmen with high negative urgency exhibit a higher error rate in the NO-GO response to phone related words, suggesting a greater implicit toward mobile phone addiction tendency, which is consistent with previous findings (Kyoung and Hyejeen, 2019). The transition from high school to university requires freshmen to adapt to new social circles, academic pressures, independent living, and the challenges of making autonomous decisions. Compared to individuals with low levels of negative urgency, those with high negative urgency seem to be more prone to emotional and impulsive reactions, responding more intensely to negative emotions and stress. They may be more likely to fall into a cycle of negative emotions, struggling to effectively cope with stress and setbacks (Guo et al., 2024), and may seek an escape from reality and a way to alleviate stress, such as using smartphones, to ease their emotional discomfort. Over time, this leads to a subconscious focus and an inability to suppress the processing of phone related words, making them more susceptible to mobile phone addiction. Conversely, individuals with low negative urgency can maintain calm and composure, better equipping them to handle the pressures of a new environment through positive means, and thus are less likely to fall into the trap of excessive smartphone use, with less processing of smartphone-related words.

The findings of Experiment 2 demonstrate a significant main effect of the negative urgency group, while the main effect of word type was not significant, consistent with the results of Experiment 1, indicating the stability and reproducibility of the impact of negative urgency on smartphone addiction. The main effect of social exclusion type is significant, and the interaction between social exclusion type and word type is also significant. In other words, under conditions of social exclusion, freshmen exhibit a higher error rate in the NO-GO response to phone related words, indicating a greater implicit mobile phone addiction tendency, which aligns with previous research (Huang and Liu, 2021; Yue et al., 2022). Lutz et al. (2023) posits that social exclusion can trigger negative emotional experiences such as anxiety, depression, loneliness, and envy in individuals, with media (mobile phone) serving as their best emotional crutch and outlet. Individuals who feel excluded can experience a sense of belonging and have their needs for control and achievement met through smartphone use, elements they may lack in real life. In essence, smartphones exert a powerful allure for those who feel excluded, an attraction they find irresistible (Leary, 1990). In such contexts, Individuals may unconsciously process mobile phone related information, as these devices offer a means of escaping reality and achieving self-worth. They may become addicted to the interaction of social media, the achievements of online games, or the sense of identity in virtual communities, which invisibly satisfy their inner needs, even if they may not be fully aware of it.

Additionally, the results revealed that there is no interaction effect between the social exclusion type and the negative urgency group. However, a significant interaction effect was found among the negative urgency group, word type, and social exclusion type. This indicates that college freshmen with varying levels of negative urgency exhibit different reactions to phone-related words and non-related words under different social exclusion scenarios, reflecting varying degrees of implicit smartphone addiction tendency. Compared to other groups, the high negative urgency-priming group displayed the highest level of implicit mobile phone addiction tendency. According to self-determination theory, an individual’s behavior is influenced by intrinsic and extrinsic motivations (Ryan and Deci, 2000). Internally, students with high negative urgency are prone to emotional instability and tension, and they may overreact to negative emotions. In such an emotional state, these students are more likely to be driven by impulses to seek immediate gratification and distraction. Externally, university freshmen often face new social environments and challenges, especially when they first adapt to college life, feeling lonely and socially excluded. Mobile phone may serve as a means of escaping reality and fulfilling social needs, leading them to engage more in virtual social interactions while neglecting real-life social opportunities. Such behavior could be perceived as implicit addiction, hindering the freshmen’s true integration into the new social environment and potentially leading to excessive dependence on smartphones. This suggests that there may be a complex interplay between negative urgency, social exclusion, and implicit mobile phone addiction tendency.

The combined effect of personality traits and the environment can influence individual behavior. This finding is significant for understanding the mental health and smartphone usage behavior of college freshmen. Further research could explore how college freshmen with different levels of negative urgency cope with social exclusion situations to reduce the mobile phone addiction tendency. This would aid in developing psychological health intervention measures tailored to university freshmen with different psychological characteristics, as well as in better understanding and preventing smartphone addiction behaviors. The study also has its limitations. Currently, there is no independent questionnaire for measuring negative urgency, and future research could develop more effective measurement tools. Extreme groups approach can compromise the statistical power and the estimation of effect sizes, thereby diminishing reliability. Future studies may employ more rigorous approaches to grouping, such as latent profile analysis, to enhance the robustness of the findings (Preacher et al., 2005). Additionally, this study only examined freshmen, and the sample lacks representativeness, which limits the further generalization of the research findings. Future studies should investigate other grades, regions, and non-university student populations to obtain more comprehensive results and conduct comparative studies.

## Data availability statement

The raw data supporting the conclusions of this article will be made available by the authors, without undue reservation.

## Ethics statement

The studies involving humans were approved by North China University of Science and Technology. The studies were conducted in accordance with the local legislation and institutional requirements. The participants provided their written informed consent to participate in this study. Written informed consent was obtained from the individual(s) for the publication of any potentially identifiable images or data included in this article.

## Author contributions

WL: Writing – review & editing, Data curation, Writing – original draft. MZ: Data curation, Writing – original draft, Investigation. RW: Writing – review & editing. MY: Writing – review & editing, Investigation. ZZ: Investigation, Writing – review & editing. SS: Writing – review & editing. LL: Writing – review & editing.
